# A cuproptosis-based prognostic model for predicting survival in low-grade glioma

**DOI:** 10.18632/aging.205834

**Published:** 2024-05-09

**Authors:** Zongren Zhao, Yuanhao Ma, Yu Liu, Zhongjun Chen, Jinyu Zheng

**Affiliations:** 1 Department of Neurosurgery, Affiliated Huaian Hospital of Xuzhou Medical University, Huaian 223002, China; 2 Department of Neurosurgery, Huzhou Central Hospital, Huzhou 313000, China; 3 Institute of Nervous System Diseases, Xuzhou Medical University, Xuzhou 221002, China

**Keywords:** LGG, cuproptosis, prognostic, immunological microenvironment

## Abstract

Background: It is unknown what variables contribute to the formation and multiplication of low-grade gliomas (LGG). An emerging process of cell death is called cuproptosis. Our research aims to increase therapeutic options and gain a better understanding of the role that cuproptosis-related genes play in the physical characteristics of low-grade gliomas.

Methods: The TCGA database was utilized to find cuproptosis genes that may be used to develop LGG risk model. Cox analysis in three different formats: univariate, multivariate, and LASSO. The gene signature’s independent predictive ability was assessed using ROC curves and Cox regression analysis based on overall survival. Use of CGGA data and nomogram model for external validation Immunohistochemistry, gene mutation, and functional enrichment analysis are also employed to clarify risk models’ involvement. Next, we analyzed changes in the immunological microenvironment in the risk model and forecasted possible chemotherapeutic drugs to target each group. Finally, we validated the protein expression levels of cuproptosis-related genes using LGG and adjacent normal tissues in a small self-case-control study.

Results: This study developed a glioma predictive model based on five cuproptosis-associated genes. Compared to the high-risk group, the low-risk group’s OS was significantly longer. The ROC curves showed high genetic signature performance in both groups. The signature-based categorisation was also linked to clinical characteristics and molecular subgroups. The prognosis of individuals with grade 2 or 3 glioma is also influenced by our risk model. Immunological testing revealed that the high-risk group had more immune cells and immunological function. The risk model also predicted immunotherapy and chemotherapy medication results. Also, this study confirmed that the expression of cuproptosis-related genes by Western blot.

Conclusion: We developed a prediction model for LGG patients using genes associated with cuproptosis. With acceptable prediction performance, this risk model may effectively stratify the prognosis of glioma patients.

## INTRODUCTION

Glioma, the most common kind of primary intracranial tumor, accounts for about 81 percent of all malignant brain tumors [1]. There is no effective treatment [2, 3]. According to the 2016 World Health Organization Classification of Tumors of the Central Nervous System [4], diffuse gliomas are described as WHO grades II and III astrocytic tumors, grade II and III oligodendrogliomas, grade IV glioblastomas, and related diffuse gliomas of childhood. Glioblastoma (WHO grade IV) is deadlier than low-grade gliomas (LGG, classes II and III) [5, 6]. Even with significant surgical resection, radiation, and temozolomide therapy, the median survival time for glioblastoma is just 16 months [7, 8]. Regardless of the development of genetic markers like IDH mutation and 1p/19q deletion, LGG diagnosis, therapy, and prognosis remain critical needs [9]. Further molecular research is needed to improve the existing diagnosis and therapy of this severe tumor.

Cell death may occur at even low levels of intracellular concentrations of copper, which is required as a cofactor for enzymes across the animal world [10]. Recent research has revealed the mechanism by which this copper-dependent cell death occurs: direct binding of copper ions to lipid acylated components of the tricarboxylic acid cycle (TCA) in mitochondrial respiration results in lipid acylated protein aggregation and subsequent downregulation of iron-sulfur cluster proteins, resulting in proteotoxic stress and, ultimately, cell death [11]. Under normal circumstances, cellular copper levels are tightly regulated to maintain proper cellular function. However, certain conditions such as disrupted copper metabolism, inflammation, or tumor development can lead to an accumulation of excess copper within cells. Excessive copper ions can interact with various molecules inside the cell, including proteins, DNA, and redox molecules. These interactions may disrupt the cellular environment, trigger a series of cell signaling pathways, and ultimately result in cell death through apoptosis. Although research on cuprotosis is still in its early stages, studies have suggested that excessive copper ions can induce apoptosis in certain types of tumors, potentially having antitumor effects. There have been no investigations of cuprotosis in tumors, and this finding is likely to lead to the creation of novel cancer therapies.

The tumor microenvironment (TME) comprises various non-cancerous cell types, including immune cells, inflammatory cells, vascular cells, fibrotic cells, and even adipocytes [12]. Cancer-promoting growth factors and cytokines are released by TAMs, increasing tumor invasion, impairing immune cell function, and encouraging angiogenesis [13, 14]. Additionally, glioma immunotherapy has been identified to cause drug resistance [15]. Exploring TAMs’ regulating mechanisms and defining the prognostic value of TAMs’ associated signature can help enhance tumor therapy.

In this research, we used cuproptosis-related genetic traits and immune response characteristics to help predict customized survival and treatment choices for individuals with LGG patients.

## MATERIALS AND METHODS

### Data collection and processing

Cuproptosis-associated genes were identified in a recent publication [8] (Supplementary Table 1). Normal brain tissue samples were obtained from the GTEx dataset (https://xenabrowser.net/datapages/). The materials utilized to build the risk model were collected from the TCGA (https://portal.gdc.cancer.gov/) database and contained gene expression and clinical information files (Supplementary Table 2). Gene expression data and clinical information files from the CGGA (http://www.cgga.org.cn/) database were utilized to validate the results (Supplementary Table 3). All of the data collected from the CGGA and TCGA sources was converted to log2(x+1) form in order to be used in future study. In order to undertake a differential analysis of cuproptosis-related genes, we used the R package “limma” (LogFC >1, FDR <0.05). We employed the R package “survival” to identify prognostic genes among those associated with cuproptosis (*P* < 0.05). The samples with missing clinical information and survival of less than 30 days were excluded, and the clinical information in the TCGA and CGGA databases utilized in this investigation was processed as indicated in Table 1.

**Table 1:** Clinical characteristics.

**Variables**	**Group**	**Number (TCGA)**	**Number (CGGA)**
Gender	Female/Male	218/262	250/339
Age	≤40/>40	233/247	306/283
Grade	G2/G3	248/232	269/230

### The risk model’s development and validation

Genes linked with prognosis and differentially expressed in grade II and III gliomas will be chosen for intersection. To design a simple and reliable model, the least absolute shrinkage and selection operator (LASSO) regression analysis was used to screen the optimum genes. With the least mean square, the best lambda and associated variables were frequently found. The following equation yields the prognostic model’s riskscore:


RiskScore=∑Exp. (Genei)×coef. (Genei)

(where Exp. (Genei) and Coef. (Genei) are the expression of Genei in a particular patient, and coefficients of the Genei in the multivariate cox regression analysis).

Patients were separated into high and low risk groups based on the median value of the risk score in order to identify TCGA patients as low or high risk. The OS of patients in various risk categories was examined using the R packages “survival” and “survminer.” The prediction effect of the model was tested using the R package “survROC” at 1, 2, 3, 4, and 5 years. PCA and t-SNE analyses were done using the R packages “Rtsne” and “ggplot2” to identify patients with varying risk levels who might be separated into two groups. Patients from the CGGA cohort were utilized to verify the model using the same approach as the TCGA cohort.

### The nomogram’s construction and evaluation

In the TCGA cohort, we used univariate analysis on clinicopathologic characteristics and our signature. Following that, the significant prognostic factors (*P* < 0.05) were included in the multivariate Cox regression analysis. The nomogram was created using the R package “rms,” and factors of predictive value in multivariate analysis (*P* < 0.05) were included. The consistency between anticipated and actual survival results was assessed using calibration curves. Nomograms, gene risk models, and clinicopathologic variables were also compared using time-dependent ROC curves.

### Protein levels of predictive signature

In the Human Protein Atlas database (HPA; http://www.proteinatlas.org/), protein expression data and immunohistochemistry findings for 32 human tissues are accessible, properly identifying the proteins in each tissue or organ. In this work, the protein expression levels of prognostic indicators in normal, low-grade, and high-grade glioma tissues were investigated utilizing HPA.

### Functional enrichment of high-and low-risk genes

The R package “limma” was used to examine differentially expressed genes in the high and low risk groups (FDR <0.05, LogFC >1). The R packages “org.Hs.eg.db,” “digest,” and “GOplot” were used to conduct GO and KEGG enrichment analysis and visualization.

### Tumor mutational burden and immune checkpoints

There are various alterations in tumor cells that make them stand out from normal cells, making them easier for the immune system to identify. As a result, patients with increased TMB should have more immunotherapeutic effects. The TCGA database was used to retrieve mutation data from Mutect software, and R was used to compute TMB. In the TCGA cohort, TMB levels were compared between high- and low-risk groups. Immune checkpoint expression was also examined between the two high-risk groups.

### Estimation of TME (tumor immune environment) cell infiltration

The proportions of different immune cells in tumor tissues and in high and low-risk groups were studied using the CIBERSORT deconvolution method in this research, and the findings were displayed in the form of bar and violin plots. The TIMER website (http://timer.cistrome.org/) was then utilized to examine the expression of immune cells such as macrophages, neutrophils, B cells, CD4+ T cells, CD8+ T cells, and dendritic cells in the high and low-risk groups. The expression levels of the 16 immune cells and 13 immunological activities among the high and low-risk groups were evaluated using a single-sample genomic enrichment analysis (ssGSEA). Heatmaps were used to visualize the findings of both studies.

### Chemotherapy and immunotherapy response prediction

Each grade II and III glioma patient’s chemotherapeutic response was predicted using the Genomics of Drug Sensitivity in Cancer public pharmacogenomic database (GDSC, http://www.cancerrxgene.org/). The R package “pRRophetic” was used to forecast drug sensitivity (IC50) values.

### Western blot

Western blotting was implemented to further verify the differential expression levels of the above genes between normal and LGG tissues. Normal brain tissues were obtained from patients with epilepsy who were undergoing temporal lobe resection. LGG tissues which were histologically diagnosed as grade II (G2), grade III (G3) was obtained from patients who received tumor resection.

The collected tissues were separately homogenized and lysed in RIPA lysis buffer containing protease and phosphatase inhibitors at 0–4°C. The homogenized protein samples were centrifuged at 1000 g for 15 min at 4°C to extract cytoplasmic proteins. The Bio-Rad protein assay kit was used to determine the protein concentration. The protein samples were homogenized with a prepared loading buffer and then boiled for 5 min at 100°C. Equal amounts of protein samples were separated through SDS-PAGE at 80 V for 1 h. Afterwards, the protein samples were transferred onto polyvinylidene difluoride (PVDF) membranes at 50 V for 1 h. The membranes were incubated for 12 h with the primary antibodies. Following this, the membranes were incubated with secondary anti-rabbit or anti-mouse horseradish peroxidase (HRP) antibodies. Ultimately, the membranes were visualized with the enhanced chemiluminescence (ECL) solution.

### Statistical analysis

Evaluation of the differential between the tumor and the surrounding tissue A *T*-test was used to examine the tissue. The overall survival rates were examined. Using the Kaplan-Meier method, overall survival prediction markers were found using univariate and multivariate Cox regression models. The R The platform is used for data statistical analysis (version 4.1.2). *P*-values less than 0.05 were regarded statistically significant (^*^P < 0.05, ^**^P < 0.01, ^***^P < 0.001, ^****^P < 0.0001).

### Data availability

This research employed normal brain tissue samples from the GTEx dataset (https://xenabrowser.net/datapages/). Gene expression datasets from glioma tissue can be obtained from the TCGA (https://portal.gdc.cancer.gov/) and CGGA (http://www.cgga.org.cn/) databases. Specific data sets can be obtained by contacting the corresponding author.

## RESULTS

### Cuproptosis-associated genes’ differential expression and prognostic analysis

**Figure 1:**
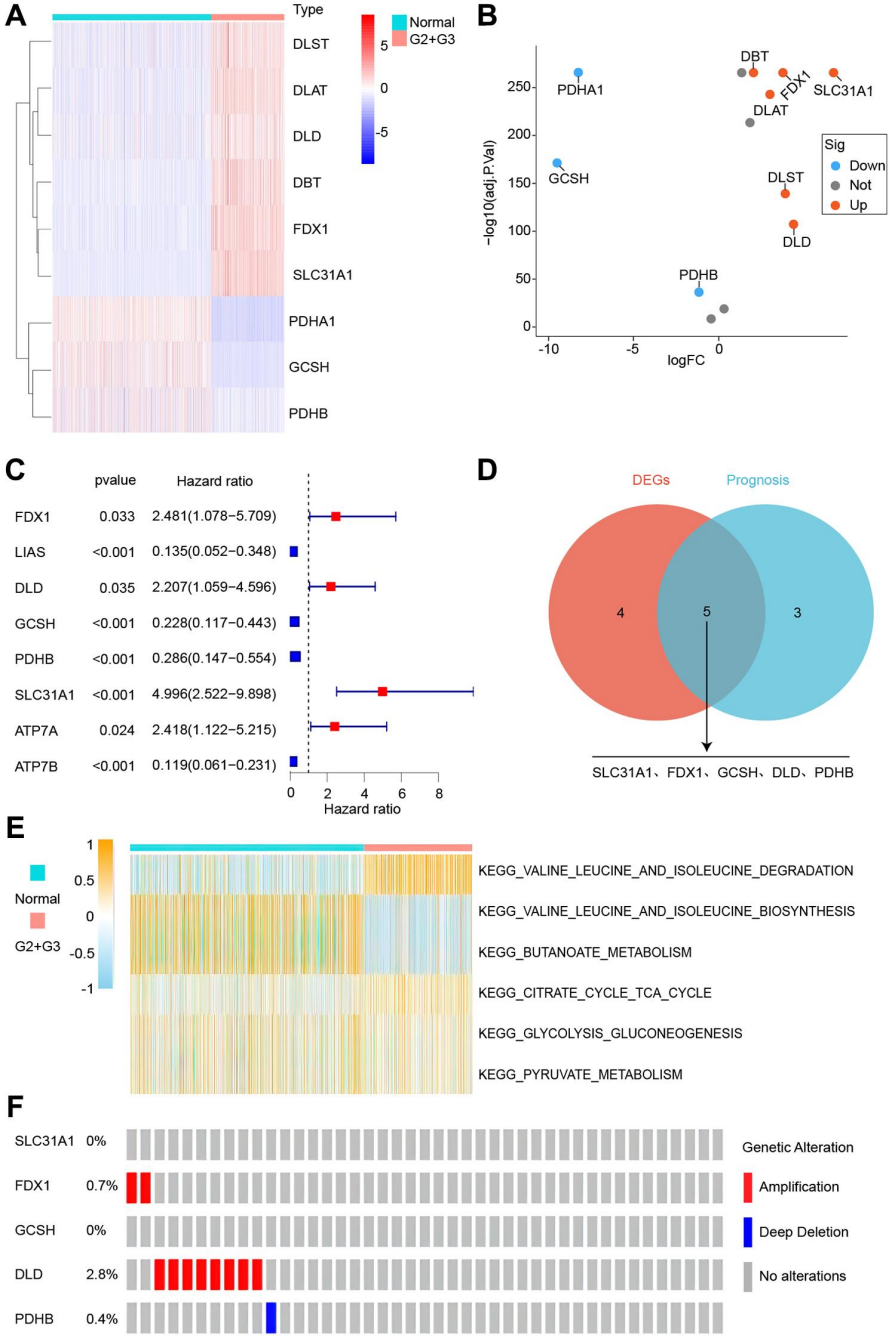
**Identification of cuproptosis-related genes.** (**A**) Gene expression heatmap. (**B**) Gene expression volcano plot. (**C**) Survival-associated genes. (**D**) Intersecting gene differences and prognosis. (**E**) Differential genes’ possible pathways. (**F**) Gene mutations analysis.

The obtained cuproptosis-associated genes were subjected to differential and prognostic analyses in normal and grade 2 and 3 glioma patients, as shown in Figure 1A, 1B, with three down-regulated and six up-regulated genes. The prognostic analysis revealed that eight genes were suitable for screening (Figure 1C). The intersection of the two genes was taken, and five overlapping genes were obtained (SLC31A1, FDX1, GCSH, DLD, PDHB, Figure 1D). As shown in Figure 1E, all six pathways that may be enriched for differentially expressed genes were investigated, and all six pathways were linked to amino acids. Finally, the mutations of SLC31A1, FDX1, GCSH, DLD, and PDHB were checked. Only DLD had the highest mutation rate of 2.8% (Figure 1F).

### Development and validation of a cuproptosis-associated gene-based glioma prognostic model

**Figure 2:**
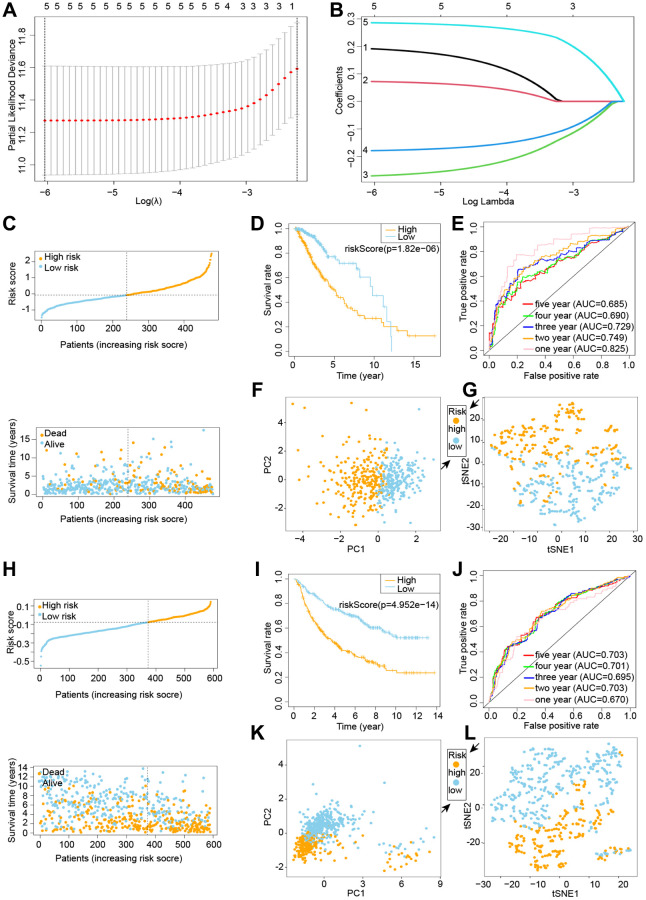
**Building and testing a cuproptosis prognostic model.** C-G denotes the TCGA cohort, whereas H-L denotes the CGGA cohort. (**A**, **B**) Visualization of LASSO regression. (**C**) Risk survival status plot. (**D**) Kaplan-Meier curve result. (**E**) The AUC of the prediction of 1, 2, 3, 4, 5-year survival rate of LGG. (**F**) PCA plot. (**G**) t-SNE plot. (**H**) LASSO regression visualization. (**I**) The outcome of the Kaplan-Meier curve. (**J**) The AUC for predicting LGG survival rates of 1, 2, 3, 4, and 5 years. (**K**) PCA plot. (**L**) t-SNE plot.

TCGA and CGGA cohorts are used in the training and testing phases, respectively, to increase the predictive model’s accuracy and precision. We identified five genes from the prior study that would be used in the next prognostic model. Cox regression analysis was used to build a gene signature using the absolute minimum shrinkage and selection operator (LASSO) (Figure 2A, 2B). We discovered that patient risk scores were adversely connected to the survival rate of glioma patients (Figure 2C). When the Kaplan-Meier curve was used for survival analysis, it revealed that the low-risk group had a much greater survival rate than the high-risk group (Figure 2D). The area under the ROC curve (AUC) for the overall survival risk score was 0.825 over one year, 0.749 over two years, 0.729 over three years, 0.690 over four years, and 0.685 over five years (Figure 2E). Patients with varied levels of risk were separated into two groups based on the findings of the PCA and the t-SNE test (Figure 2F, 2G).

We exploited the CGGA cohort as a validation group for the prognostic model that was developed by the TCGA cohort. We divided the calculated risk scores into high-risk and low-risk groups based on median values, with patients in the high-risk group having a higher probability of early death than patients in the low-risk group (Figure 2H). The survival curves revealed that the low-risk group had a much greater survival rate than the high-risk group (Figure 2I). The ROC curve reveals an AUC of 0.670 in 1 year, 0.703 in 2 years, 0.695 in 3 years, 0.701 in 4 years, and 0.703 in 5 years (Figure 2J). The PCA and t-SNE results suggested that patients with varying risks were well split into two groups (Figure 2K, 2L).

Finally, we used the TCGA and CGGA datasets to conduct survival and ROC analyses on each of these five genes in glioma (Table 2). These findings suggest that our risk model outperforms single genes in terms of survival prediction.

**Table 2:** Survival prediction using genetic and risk models.

**Database**	**Gene**	**Survival (*p*-value)**	**ROC (AUC value)**				
			**One year**	**Two years**	**Three years**	**Four years**	**Five years**
TCGA	SLC31A1	9.667e−04	0.76	0.702	0.679	0.612	0.594
	FDX1	0.021	0.591	0.629	0.624	0.614	0.603
	GCSH	0.001	0.223	0.339	0.345	0.351	0.354
	DLD	0.086	0.587	0.584	0.605	0.564	0.592
	PDHB	6.601e−04	0.262	0.350	0.400	0.374	0.339
	riskScore	1.82e−06	0.825	0.749	0.729	0.690	0.685
CGGA	SLC31A1	5.039e−06	0.599	0.622	0.606	0.604	0.607
	FDX1	1.548e−06	0.53	0.598	0.611	0.63	0.643
	GCSH	1.658e−04	0.36	0.377	0.382	0.385	0.393
	DLD	4.266e−04	0.523	0.57	0.558	0.575	0.587
	PDHB	0.182	0.5	0.505	0.519	0.516	0.504
	riskScore	4.952e−14	0.67	0.703	0.695	0.701	0.703

### Risk model-clinical trait relationships

**Figure 3:**
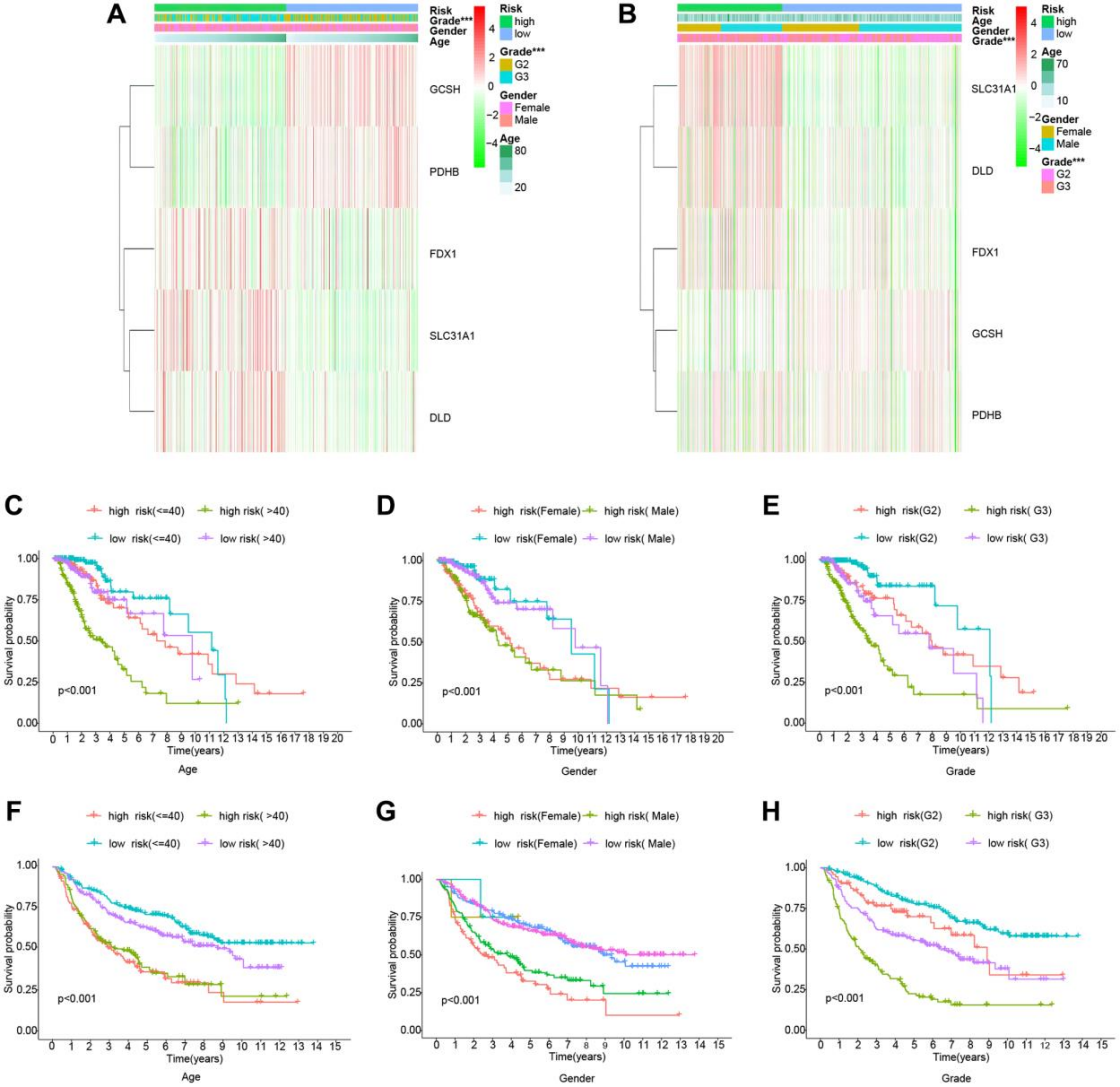
**Clinically relevant heatmaps and survival analysis of subtypes of clinical features.** (**A**) Clinical relevance heatmap for the TCGA cohort. (**B**) Clinical relevance heatmap for the CGGA cohort. (**C**–**E**) The TCGA cohort's survival analysis. (**F**–**H**) The CGGA cohort’s survival analysis.

**Figure 4:**
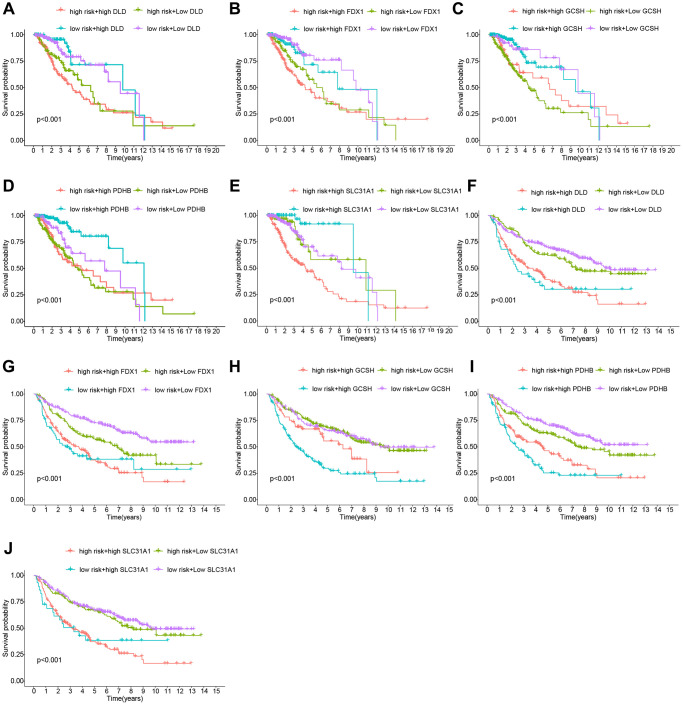
**Analysis of the risk Scores and genes for survival.** (**A**–**E**) Cohort of the TCGA. (**F**–**J**) Cohort of the CGGA.

To further evaluate the risk model’s dependability, we compared the risk score to individual clinical features from the TCGA and CGGA cohorts. In the TCGA cohort, only tumor grade was connected with the risk score substantially, and GCSH and PDHB were favorably associated with the risk score, but FDX1, SLC31A1, and DLD were negatively associated with the risk score (Figure 3A). The CGGA cohort study provided further support for the TCGA findings (Figure 3B). Following that, we ran a survival study on the risk model’s subtypes of clinical features. Survival results by age, gender, and tumor grade were comparable to the overall survival outcome in the TCGA cohort (Figure 3C–3E). By studying the CGGA cohort (Figure 3F–3H), we achieved similar findings with the TCGA cohort. Thus, our risk model demonstrated some predictive value for survival in terms of clinical feature subtypes. Finally, we conducted a survival analysis on the levels of expression of different genes in the risk model. The TCGA dataset is shown in Figure 4A–4E, whereas the CGGA dataset is represented in Figure 4F–4J. All of these findings reinforce the notion that our model has a high prediction ability for survival.

### Nomogram construction and assessment

**Figure 5:**
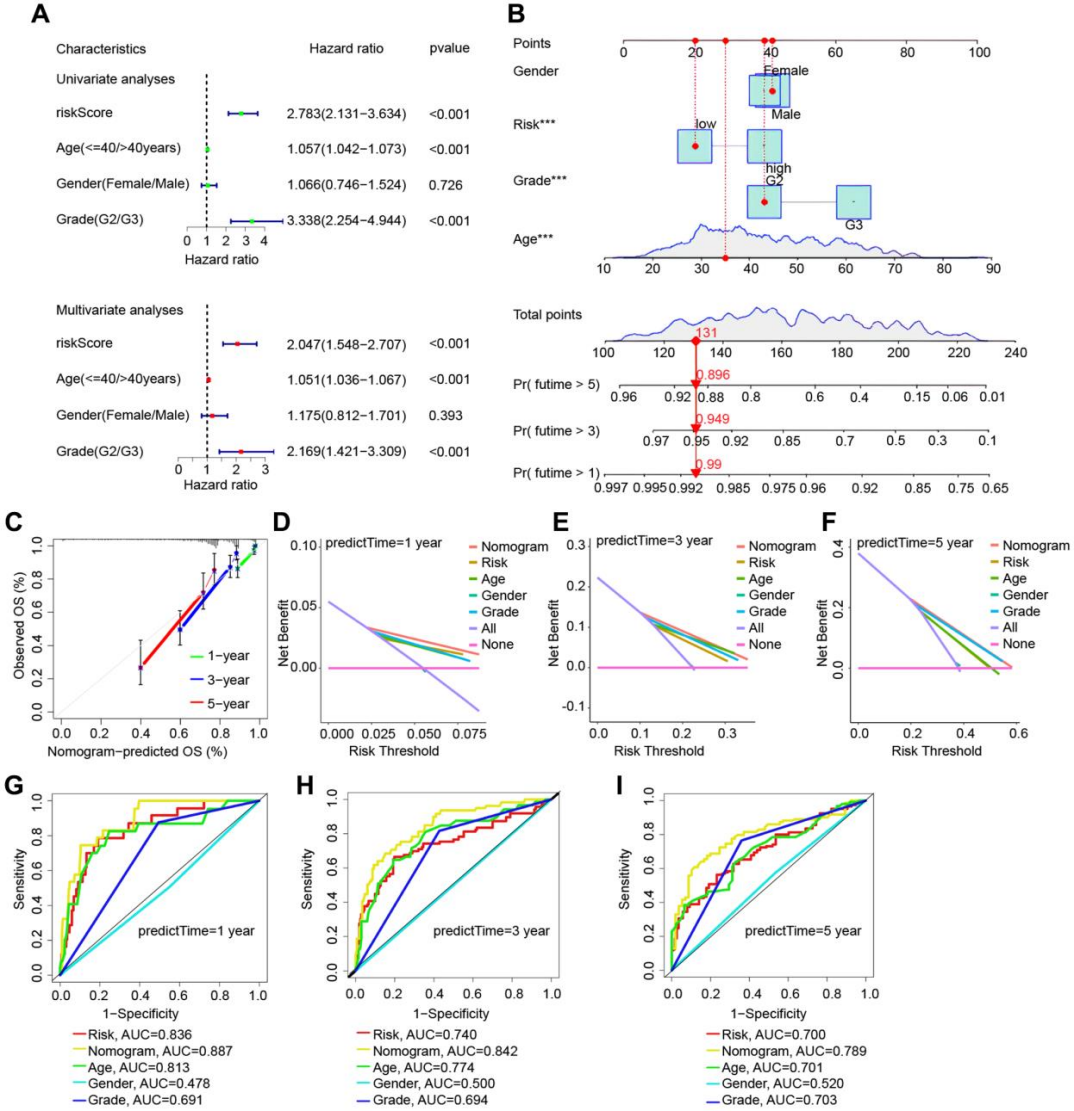
**Validation of the risk model based on the TCGA cohort’s nomogram model.** (**A**) Univariate and multivariate Cox regression forest plots in LGG. (**B**) Nomogram for LGG sufferers’ OS. (**C**) Calibration plot of the nomogram for OS probability prediction at 1, 3, and 5 years. (**D**–**F**) 1, 3, 5 years decision curve. (**G**–**I**) ROC curves for 1-, 3-, and 5-years survival prediction.

In the TCGA cohort, univariate and multivariate Cox regression studies found certain clinical features associated with glioma prognosis (Figure 5A). A nomogram was created based on several independent prognostic criteria, including riskscore, age, gender, and grade, to increase prediction capacity and give a quantitative tool for predicting the survival outcomes of glioma patients in clinical practice (Figure 5B). Calibration curves indicated that there was an excellent match between the actual probability of 1-year, 3-year, and 5-year OS and the anticipated probability of the nomogram (Figure 5C). We also used decision curves and ROC curves for risk scores and clinical features to predict survival at 1, 3, and 5 years. Figure 5D–5F show the decision curves for 1, 3, and 5 years, respectively, while Figure 5G–5I depict the ROC curves for 1, 3, and 5 years. All of these analytical methodologies demonstrate that our risk model has strong survival prediction ability.

### Immunohistochemistry testing of five genes in the risk model

**Figure 6:**
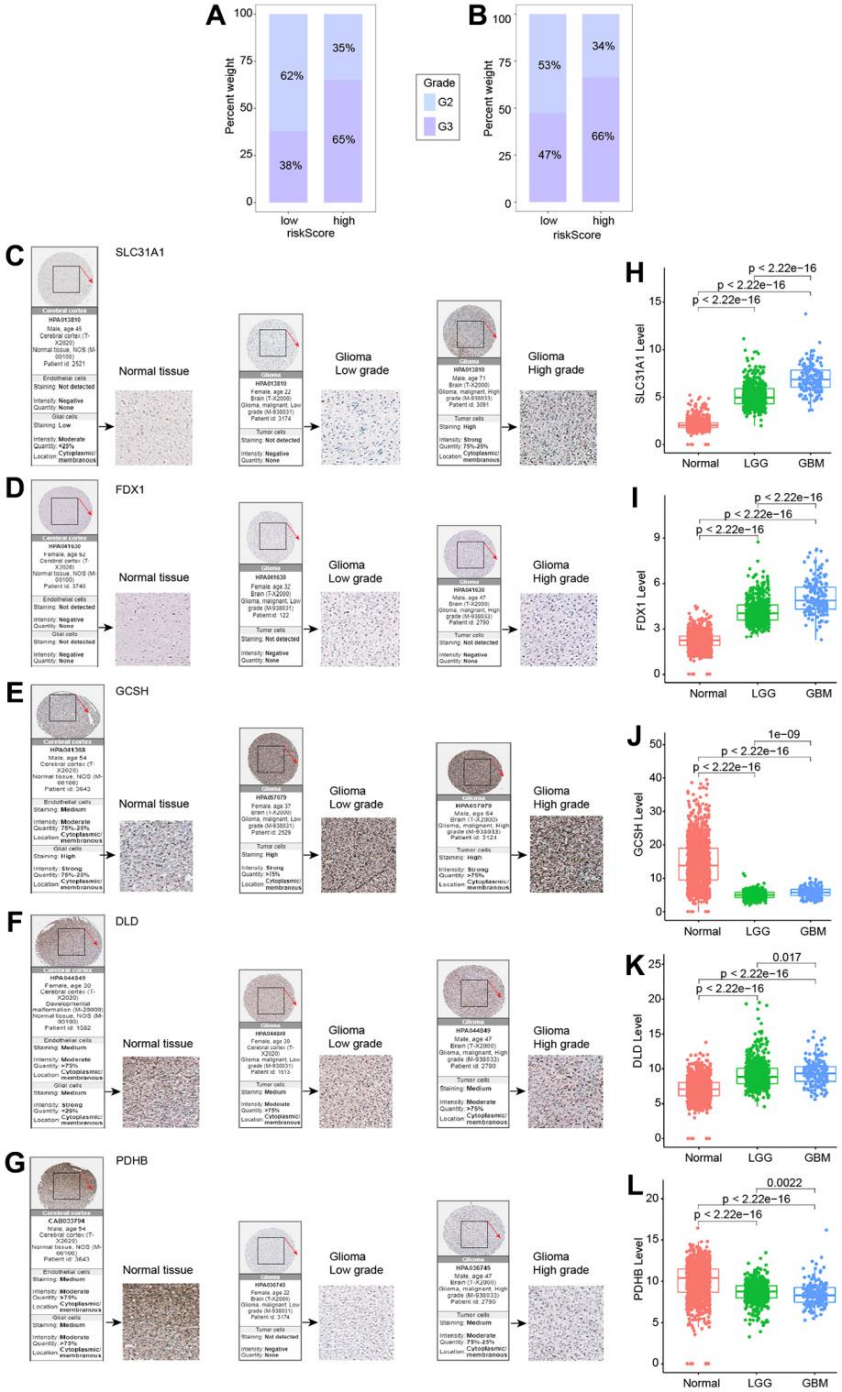
**Expression of five genes in different tumor grades.** (**A**) G2 and G3 distribution in high- and low-risk groups in the TCGA cohort. (**B**) G2 and G3 distribution in high- and low-risk groups in the CGGA cohort. (**C**–**G**) Five genes examined via IHC. (**H**–**L**) Expression of five genes in normal, LGG, and GBM.

The percentage of G2 and G3 in the high and low risk groups in the TCGA (Figure 6A) and CGGA (Figure 6B) datasets was initially examined. G2 was more prevalent in the low-risk group, whereas G3 was more prevalent in the high-risk group. Subsequently, we utilized the HPA database and data from the TCGA and GTEx datasets to run a bioinformatics study to verify the expression of the five genes used to build the risk model in glioma patients. All of the data indicated that SLC31A1, FDX1, and DLD protein expression levels rose with tumor grade, whereas GCSH and PDHB protein expression levels dropped (Figure 6C–6L).

### Gene enrichment analysis of cuproptosis-related genes

**Figure 7:**
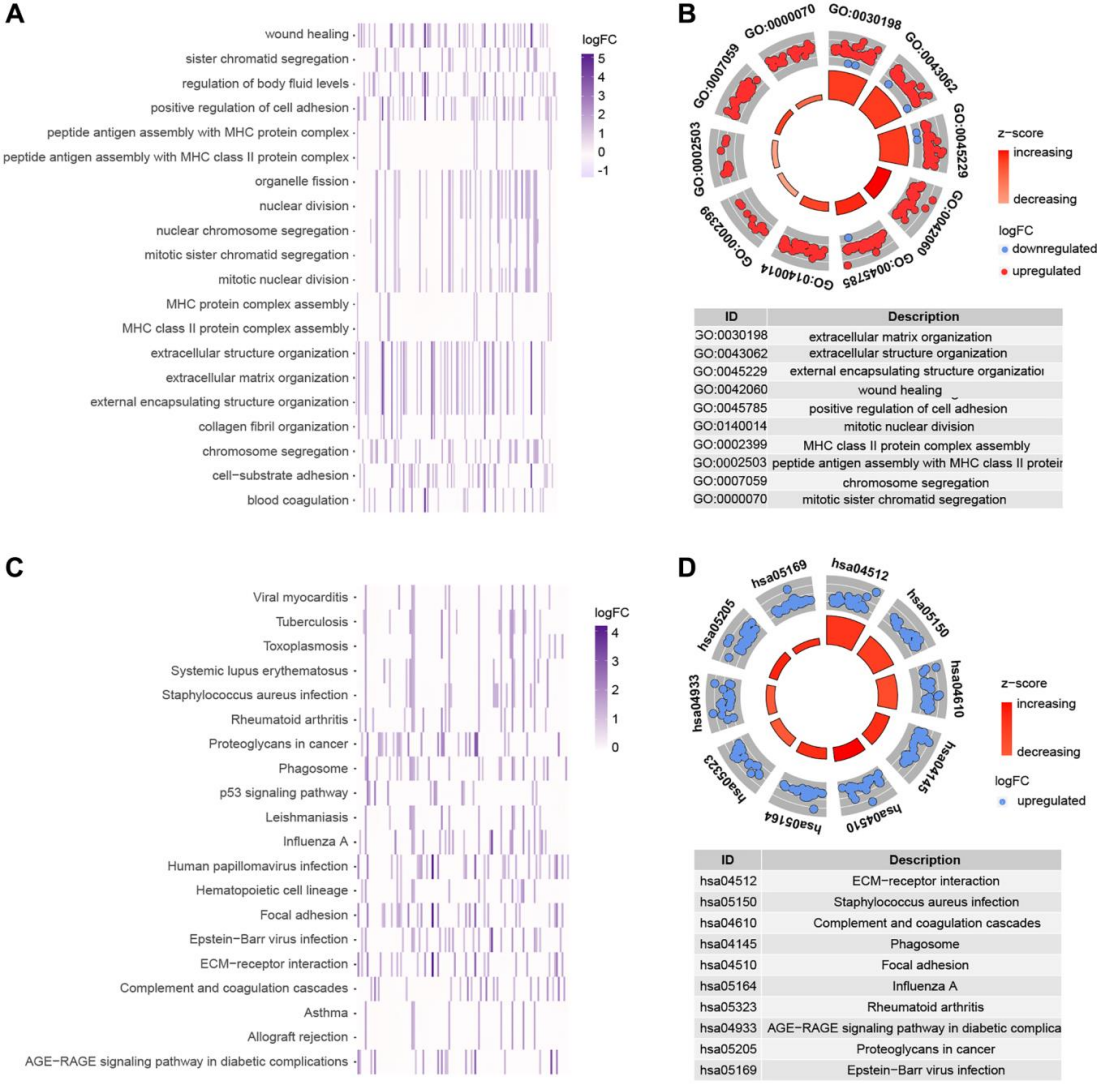
**Functional analysis of genes that are differently expressed in high and low risk groups.** (**A**, **B**) GO enrichment analysis. (**C**, **D**) KEGG enrichment analysis.

This research employed GO enrichment analysis and KEGG pathway analysis of differentially expressed cuproptosis-related genes between high-risk and low-risk groups to investigate the biological roles and processes of genes linked with risk scores. We discovered 843 potential pathways in the GO enrichment study, including BP, CC, and MF (Supplementary Table 4). Figure 7A, 7B depicts several of the top-ranked pathways, which are mostly connected to extracellular matrix and structure, cell adhesion, and chromosomes. We enriched 50 pathways (Supplementary Table 5) in the KEGG enrichment analysis, most of which included ECM-receptor interactions and complement and coagulation cascades, among other things (Figure 7C, 7D).

### Analysis of gene correlations and mutations

**Figure 8:**
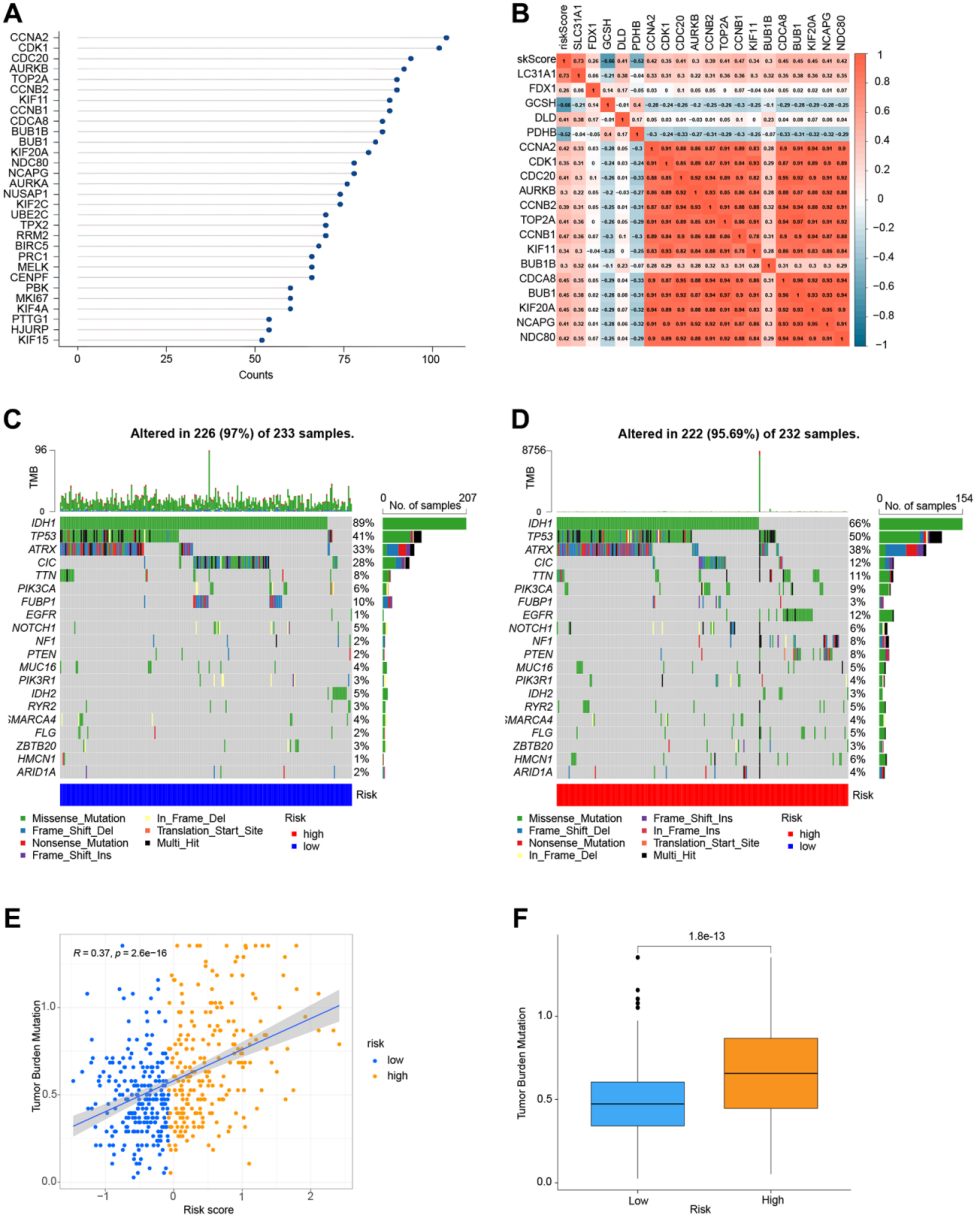
**Correlation and mutation analysis of genes.** (**A**) The number of interactions between proteins. (**B**) Analysis of the correlation between genes and risk scores. (**C**) Mutation analysis of genes in the low-risk group. (**D**) Mutation analysis of genes in the high-risk group. (**E**) TMB and risk score co-expression analysis. (**F**) Analysis of TMB in high-and low-risk groups.

We used the STRING website to conduct a functional protein association network analysis of the differentially expressed genes in high-and low-risk groups (Supplementary Table 6). By measuring the number of protein-protein interactions each gene has (Figure 8A), we were able to identify the top 30 genes with the most protein-protein interactions. An investigation of the relationships between the top 14 gene candidates, five genes linked to cuproptosis, and the risk score is shown in Figure 8B. As a follow-up, we examined the mutations of genes in the high and low-risk groups. For the low-risk and high-risk groups, respectively, Figure 8C, 8D show the top 20 genes with the greatest mutation frequency. Finally, co-expression relationships between tumor mutational burden and risk score (Figure 8E) and their expression in the high and low-risk groups were investigated (Figure 8E, 8F).

### Immunization and the risk model

**Figure 9:**
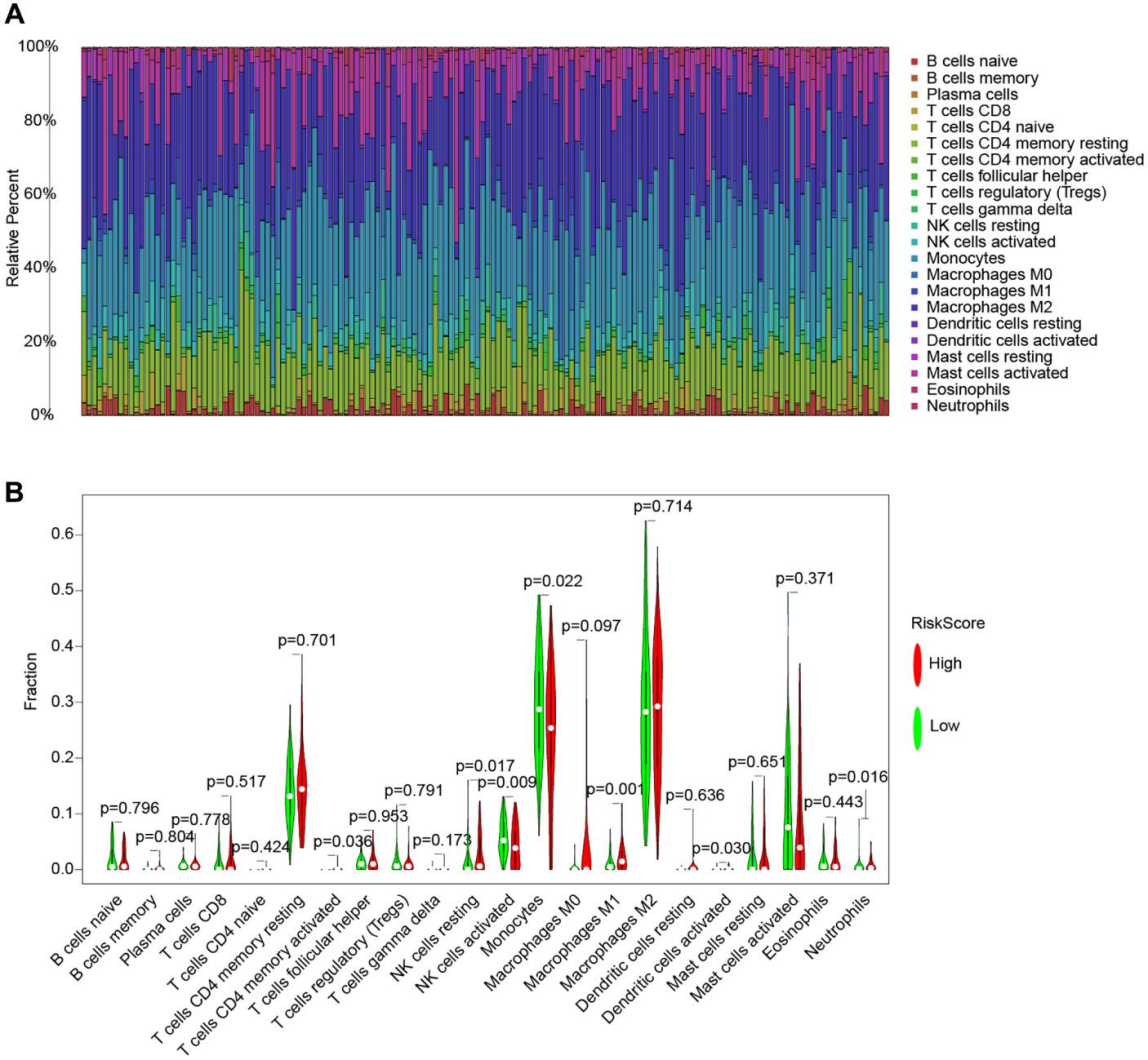
**The CIBERSORT method for immune cell and risk model analysis.** (**A**) A histogram of the percentage of 22 immune cell species in TCGA samples. (**B**) Violin plot of the percentage of immune cells in the sample.

Conventional treatment for gliomas, which have a high risk of recurrence and aggressiveness, is not effective. The purpose of this study was to see whether our risk models might be used to guide glioma immunotherapy based on their correlation with immune infiltration. Three approaches, CIBERSORT, TIMER, and ssGSEA, are used to examine the connection between risk models and vaccination in this research. Figure 9 depicts the association between immune cells and the risk model using the CIBERSORT approach. Figure 9A shows the expression of immune cells in each TCGA cohort sample. The violin plot then visualizes the findings of the statistical analysis of immune cell infiltration in the high-and low-risk groups (Figure 9B).

**Figure 10:**
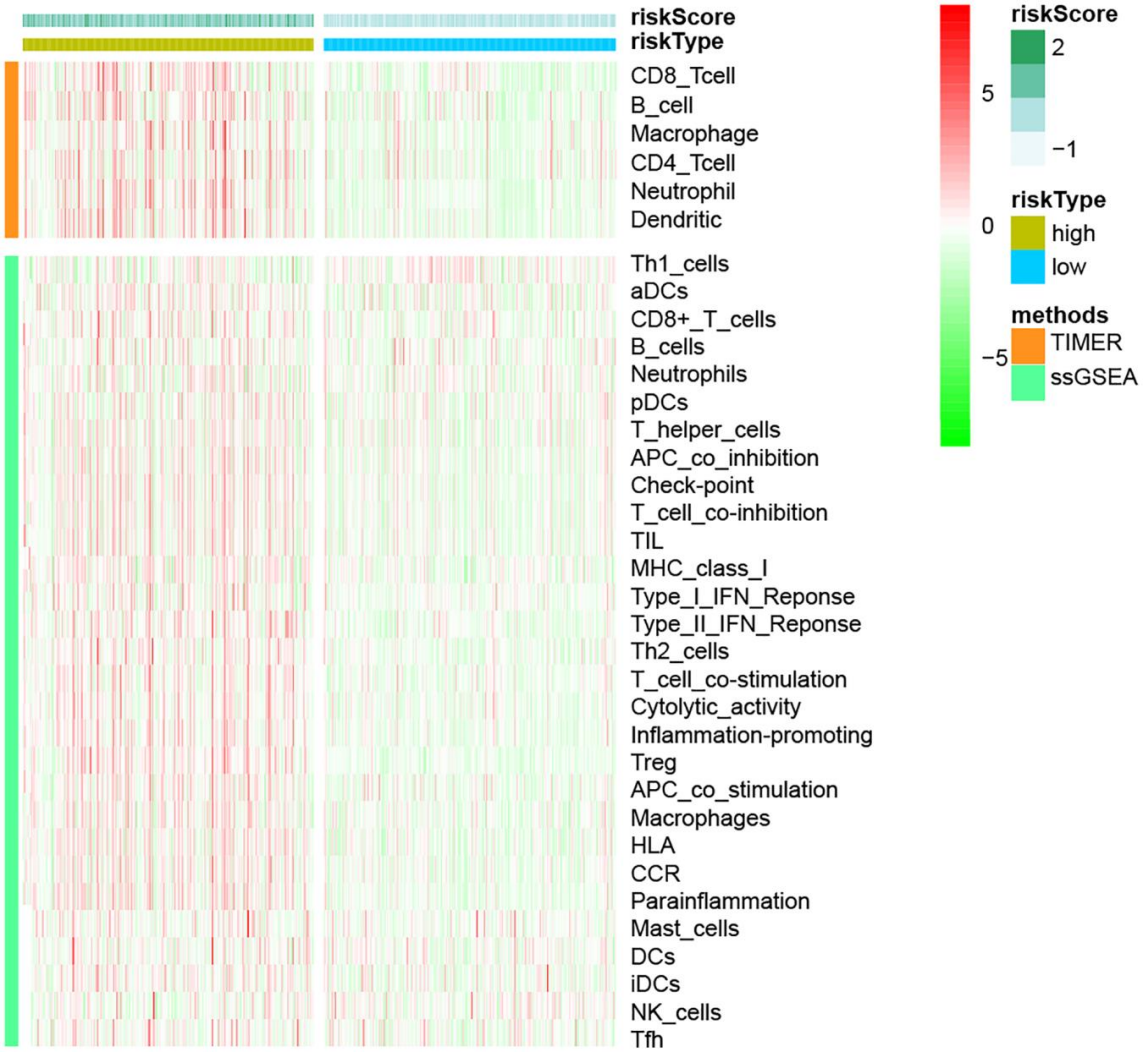
The link between immune cell infiltration and risk models, based on TIMER and ssGSEA methodologies.

Following that, we used the TIMER and ssGSEA methodologies to explore the relationship between immune cells and immunological function and risk scores (Figure 10). Using the TIMER method, we discovered that in the high-risk group, all six immune cell types were increased. We used the ssGSEA method to examine the expression of 16 immune cells and 13 immunological functions in species from the high-and low-risk groups. The findings revealed that the majority of them were elevated in the high-risk group.

### Immune checkpoint blockade (ICB) and chemotherapeutic sensitivity

**Figure 11:**
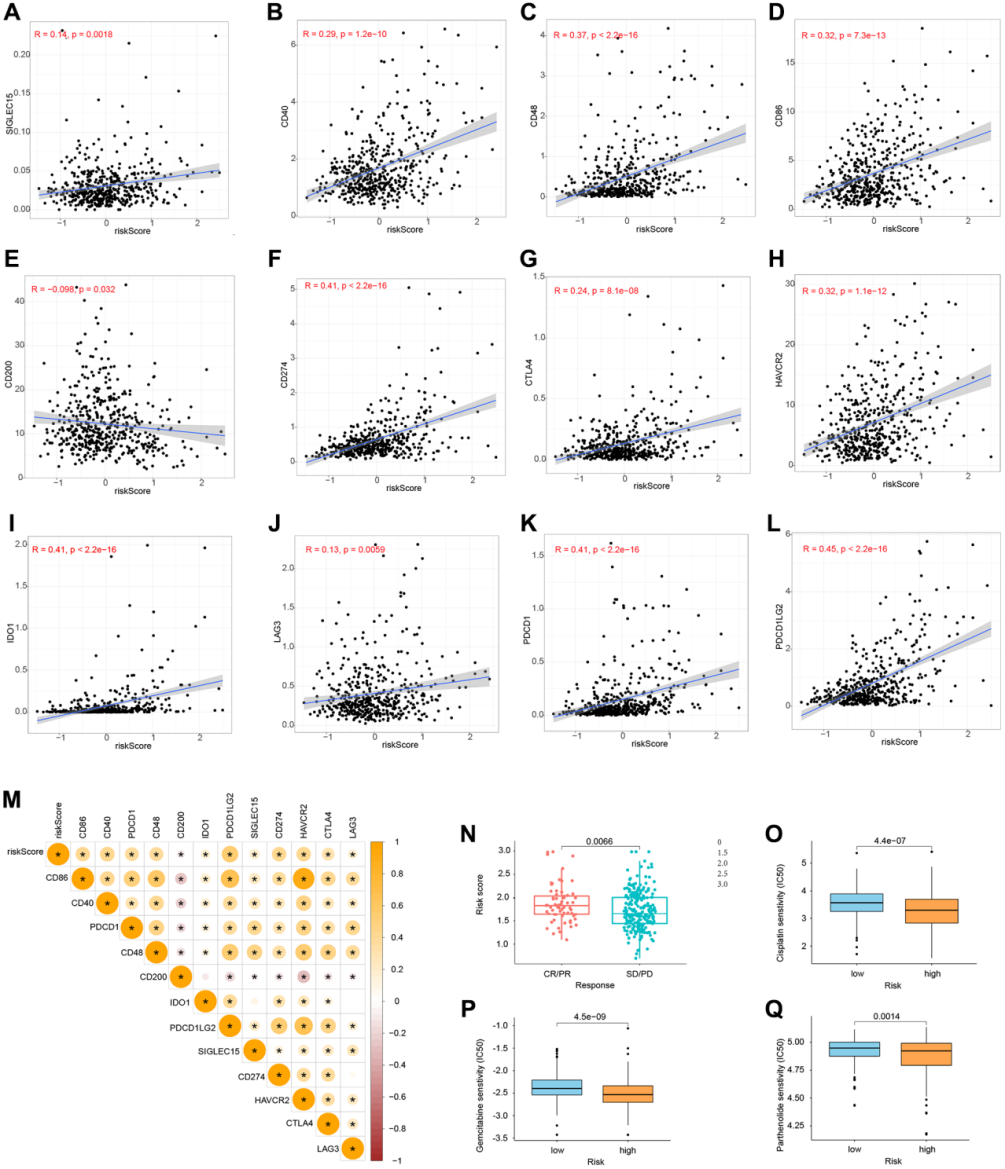
**Immune checkpoints and chemotherapy sensitivity.** (**A**–**M**) Relationship between risk model and immunological checkpoints. (**N**) Immunotherapy response and risk score. (**O**–**Q**) Chemotherapy sensitivity and risk score.

We conducted a correlation study between the expression levels of 12 essential genes and risk scores in ICB to investigate the potential relevance of risk models in the treatment of ICB in grade II and grade III gliomas. The findings revealed that the risk score was highly associated with all 12 important genes (Figure 11A–11M). Next, we looked at the response of immunotherapy in high- and low-risk groups, respectively. For individuals with a high risk of complications, immunotherapy improved their outcomes (Figure 11N). Finally, we discovered three medicines (Cisplatin, Gemcitabine, and Parthenolide) that may be employed in glioma chemotherapy. A high-risk score was shown to be related to a lower half-inhibitory concentration (IC50) of chemotherapeutic medications (Figure 11O–11Q). This demonstrated that the model may be used to predict chemosensitivity.

### Western blot

**Figure 12:**
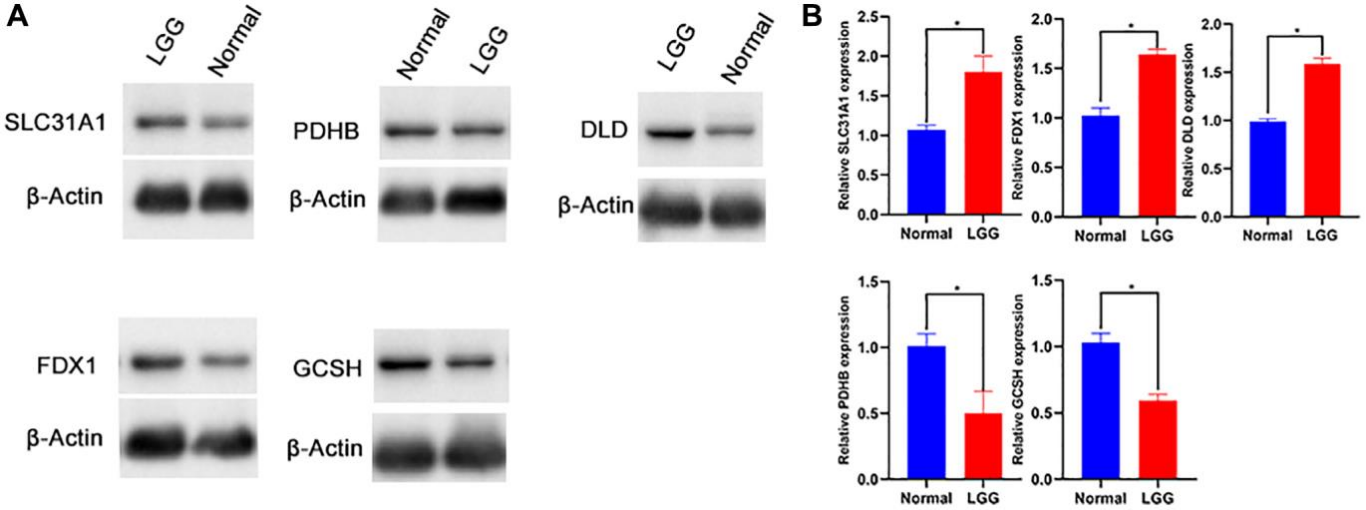
Western blot (**A**, **B**) of SLC31A1, FDX1, DLD, GCSH, PDHB protein.

The protein expression level of SLC31A1, FDX1, DLD, GCSH, PDHB protein was confirmed. The results showed that compared with normal tissues, the protein expression level of SLC31A1, FDX1, DLD was higher. Whereas GCSH, PDHB protein was lower (Figure 12A, 12B).

## DISCUSSION

LGG is more common among the elderly. People who are younger (on average, 41 years old) have a better chance of surviving, with a median survival duration of seven years [16]. Resistant cancer and recurrence are unavoidable, despite significant advancements in neurosurgery and other forms of treatment, including chemotherapy and radiation [17]. Malignancy has great variation as a result of biological processes. Some patients have indolent fates, whereas others acquire high-grade gliomas with unpleasant consequences [18]. However, despite the fact that LGG is considered less aggressive, patients often have a wide range of survival results [19]. As a result, current research is focused on developing new indicators that may accurately predict patients’ prognoses.

The genetic and molecular heterogeneity of LGG affects the effectiveness of immunotherapies [20]. The LGG immune microenvironment is a complicated neuroinflammatory network that includes both positive and negative immune regulators [21]. Copper induces cell death by a mechanism known as protein lipid acylation, which is mostly seen in the TCA cycle [8, 22, 23]. Currently, no research on cuproptosis-related genes in malignancies has been published. If a full investigation of its associated genes in glioma can be undertaken, it may lead to more successful customized glioma treatment techniques. Here, we created a risk model that may help determine the prognosis of patients with low-grade gliomas and provide information about those tumors’ immune characteristics. Additionally, the results from this study may potentially serve as a useful resource for guiding the treatment of low-grade glioma.

We formulated a risk model for the disease based on five cuproptosis-associated genes: SLC31A1, FDX1, GCSH, DLD, and PDHB. Increased messenger RNA (mRNA) levels in colorectal carcinogenesis have been found in human solute carrier family 31 member 1 (SLC31A1), a copper transporter 1 (CTR1), a homologous high-affinity plasma membrane copper transporter that impacts dietary copper absorption [24, 25]. Exosomal miR-375 from hMSCs has been found to impede the development of glioma cells by inhibiting SLC31A1, making it a viable target for the treatment of gliomas [26]. Elesclomol induces a novel kind of copper-dependent cell death, which is enhanced by the ferredoxin 1 (FDX1) protein, which binds and lowers the elesclomol–Cu (ii) complex. This novel method of cell death demonstrates how a change in energy metabolism may be used to enhance the efficiency of a cancer targeting medication and hence prevent cancer cells from adapting to proteasome inhibition and developing treatment resistance [27]. The findings demonstrated that FDX1 knockdown mostly enhanced glycolysis and fatty acid oxidation while altering amino acid metabolism, offering new insights into the oncogenic function of FDX1 in LUAD [28]. GCSH has the potential to evaluate the viability of breast cancer cells [29] and is a candidate gene for non-ketotic hyperglycemia [30–32]. For the time being, no studies of GCSH in glioma have been reported. As an autoantigen unique to endometrial cancer patients, dihydrolipoamide dehydrogenase (DLD) has been discovered in mitochondria. Endometrial cancer may be diagnosed using IgA autoantibodies against DLD [33]. The DLD genotype appears to elevate the risk of Alzheimer’s disease, independent of APOE [34]. MiR-3663-3p levels were shown to be considerably greater in glioma tissues, where they increased cell proliferation, protected against apoptosis, and accelerated invasion by directly targeting PDHB [35]. Pseurotin A has the ability to target a number of metabolic enzymes (including PDHB) in the fight against glioma [36]. In addition, PDHB has been associated with the development of several diseases [37–40].

Through GO enrichment analysis and KEGG pathway analysis, we have identified close associations between copper death-related genes and the following pathways, Extracellular matrix (ECM) and structure: The ECM plays a crucial role in providing structural support to cells and tissues. It is involved in various cellular processes such as cell migration, proliferation, and differentiation. Enriched pathways related to the ECM may include signaling pathways involved in ECM remodeling, cell-matrix interactions, and tissue development. Cell adhesion: Cell adhesion pathways are involved in mediating the physical attachment between cells and their surrounding environment. These pathways regulate processes such as cell migration, tissue morphogenesis, and immune response. Enriched pathways may include adhesion molecule signaling, integrin-mediated signaling, and cytoskeletal remodeling. Chromosomes: Chromosomal pathways are associated with the organization, replication, and maintenance of DNA within the nucleus. Enriched pathways related to chromosomes could involve DNA replication and repair mechanisms, mitotic cell division, and epigenetic regulation. By exploring these enriched pathways, we may gain insights into the molecular mechanisms underlying the involvement of cuproptosis in various biological and pathological contexts.

Although our research has established a model for evaluating the prognosis of glioma, there are certain limitations to our work. More samples are needed, and we need to keep testing the model to see if it needs any tweaks. One method of cell death is via the process of cuproptosis. Further research on the mechanism of cuproptosis induction is required. It’s encouraging to know that this model, which uses gene expression to predict survival rates, might give an innovative approach to the search for more effective LGG therapy options.

## CONCLUSIONS

With the use of five cuproptosis-associated genes, we were able to create and verify a predictive model, which may be a predictor of response to LGG patients and immunotherapy. Despite its retrospective nature limiting the breadth of our research, additional experimental investigations are necessary to consolidate these insights and elucidate the underpinning processes. In conclusion, the goal of this research was to create a predictive model related to cuproptosis that could be used to predict survival in LGG patients on an independent basis. Moreover, it holds potential in shaping treatment strategies by gauging responsiveness to immune checkpoint inhibitors and chemotherapeutic agents, among other interventions.

## Supplementary Materials

Supplementary Table 1

Supplementary Table 2

Supplementary Table 3

Supplementary Table 4

Supplementary Table 5

Supplementary Table 6
