# Supplementary material for: A cuproptosis-based prognostic model for predicting survival in low-grade glioma
Source: Aging (Albany NY). 2024 May 9;16(10):8697–716. doi: 10.18632/aging.205834 (PMC11164498; doi:10.18632/aging.205834)
Supplement: Supplementary Table 1 [file aging-16-205834-s001.pdf]

SUPPLEMENTARY TABLE

Supplementary Table 1. Gene for cuproptosis.

FDX1
LIPT1
LIAS
DLD
DBT
GCSH
DLST
DLAT
PDHA1
PDHB
SLC31A1
ATP7A
ATP7B
